# Transdermal Hormonal Therapy in Menopause: Current Evidence and Personalized Approaches

**DOI:** 10.3390/pharmaceutics18050529

**Published:** 2026-04-27

**Authors:** Mara-Mădălina Mihai, Ana-Maria Toma, Cristian-Valentin Toma, Andra-Ioana Copilău, Cătălina-Ioana Naum, Maria-Alexandra Timofte, Ileana-Adela Văcăroiu, Andra-Elena Balcangiu Stroescu, Romina Marina Sima, Mircea-Octavian Poenaru

**Affiliations:** 1 Department of Oncologic Dermatology, “Elias” Emergency University Hospital, “Carol Davila” University of Medicine and Pharmacy, 020021 Bucharest, Romania; mara.mihai@umfcd.ro (M.-M.M.); andra-ioana.copilau@rez.umfcd.ro (A.-I.C.); catalina-ioana.naum@rez.umfcd.ro (C.-I.N.); maria-alexandra.timofte@rez.umfcd.ro (M.-A.T.); 2 Department of Urology, “Professor Doctor Theodor Burghele” Clinical Hospital, “Carol Davila” University of Medicine and Pharmacy, 020021 Bucharest, Romania; cristian.toma@umfcd.ro; 3 Department of Nephrology, “Sf. Ioan” Clinical Emergency Hospital, Faculty of Medicine, “Carol Davila” University of Medicine and Pharmacy, 020021 Bucharest, Romania; ileana.vacaroiu@umfcd.ro; 4 Discipline of Physiology, Faculty of Dental Medicine, “Carol Davila” University of Medicine and Pharmacy, 020021 Bucharest, Romania; andra.balcangiu@umfcd.ro; 5 Department of Obstetrics and Gynecology, “Carol Davila” University of Medicine and Pharmacy, 020021 Bucharest, Romania; romina.sima@umfcd.ro (R.M.S.); mircea.poenaru@umfcd.ro (M.-O.P.); 6 “Bucur” Maternity, Saint John Hospital, 012361 Bucharest, Romania

**Keywords:** female hormonal health, transdermal hormone therapy, estrogen, progesterone, testosterone, menopause, personalized medicine, transdermal delivery

## Abstract

Maintaining hormonal equilibrium is a key determinant of women’s health, particularly during the menopausal transition and postmenopause. The decline in ovarian estrogen and progesterone production influences multiple physiological systems, affecting many aspects like vasomotor stability, bone and cardiovascular health, cognitive function, mood, and metabolic regulation. As a result, many women may experience symptoms that impair daily functioning and increase long-term morbidity. Recent progress in menopausal care emphasizes individualized, evidence-guided treatment, supported by improved diagnostic tools that allow for a more precise assessment of endocrine changes during this life stage. Among the available therapeutic options, transdermal menopausal hormone therapy has gained growing recognition due to its ability to re-establish hormonal levels with fewer systemic effects. By bypassing first-pass hepatic metabolism, this route provides more consistent serum hormone concentrations and may be associated with a lower risk of metabolic and thromboembolic complications compared with oral formulations. This review brings together the physiological basis, clinical indications, and current scientific evidence related to transdermal hormonal therapy during menopause while also highlighting its expanding therapeutic role and integration into personalized treatment strategies. In addition, we discuss recent findings on its pharmacological profile, clinical effectiveness, and emerging perspectives that position this therapeutic option as an increasingly important component of modern menopausal care and women’s health management.

## 1. Introduction

Female hormonal health is a key element of physical, emotional, and reproductive well-being throughout a woman’s life. Numerous physiological functions, such as menstrual cycle regulation, fertility, bone density, cardiovascular and cognitive function, mood stability, and metabolic balance, are regulated by hormones like estrogen, progesterone, and androgens [1,2]. Whether caused by pathological conditions like polycystic ovary syndrome (PCOS), hypothalamic amenorrhea, or premature ovarian insufficiency, or physiological changes like puberty, pregnancy, and menopause, disturbances in hormonal homeostasis can have a substantial negative influence on quality of life and long-term health outcomes [3].

The need for customized, evidence-based strategies for women’s hormonal health has become increasingly evident in recent years. Our understanding of endocrine fluctuations across every stage of life has improved thanks to developments in hormone monitoring and diagnostic tools. At the same time, treatment options for hormonal imbalances have advanced, with a focus on physiological relevance, safety, and efficacy [3,4]. Although serum follicle-stimulating hormone, estradiol, and anti-Müllerian hormone levels are commonly used to support the assessment of menopausal status, their clinical utility is limited by substantial interindividual variability and hormonal fluctuations during the perimenopausal transition. Single measurements may not accurately reflect ovarian reserve or symptom severity. Consequently, menopause remains primarily a clinical diagnosis based on menstrual history and symptomatology, with laboratory testing serving as a complementary rather than definitive tool.

Among these, transdermal hormone treatment has drawn interest due to its capacity to minimize systemic risks while restoring hormonal balance. Over the past decade, this therapeutic preference has translated into a striking rise in clinical use. Large population-based analyses show that prescriptions for transdermal hormone therapy have increased more than tenfold, reflecting both growing physician confidence and patient acceptance. This steady upward trend, observed across multiple European health systems, signals a paradigm shift in menopausal management toward delivery methods that combine efficacy with improved long-term tolerability, which directly influences quality of life, treatment acceptance, and adherence [4,5].

This shift underscores a broader movement toward individualized hormone therapy, in which treatment decisions are guided not only by symptom control but also by risk stratification and long-term safety considerations. Contemporary population studies from Europe highlight that compared to oral administration, this delivery method avoids hepatic first-pass metabolism, enables steady-state hormone levels, and may have a lower risk profile for metabolic and thromboembolic consequences [4,5]. With continuous advances in medical research and treatment technologies, transdermal hormone therapy has emerged as a significant innovation in menopausal management.

Recent advances in transdermal drug delivery technologies and neuroendocrine research have further refined the understanding of menopausal hormone therapy. Innovative delivery platforms, including nanostructured and controlled-release systems, have demonstrated improved pharmacokinetic stability and tissue targeting, potentially enhancing therapeutic efficacy and safety. In parallel, emerging neurobiological studies have provided deeper insight into the role of estrogen in brain aging and cognitive resilience during menopause. These developments support the growing emphasis on personalized and mechanism-based approaches to menopausal hormone therapy [6,7,8].

This review brings together the physiological basis, clinical indications, and current scientific evidence related to transdermal hormone therapy during menopause while also highlighting its expanding therapeutic role and integration into personalized treatment strategies. In addition, we discuss recent findings on its pharmacological profile, clinical effectiveness, and emerging perspectives that position this therapeutic option as an increasingly important component of modern menopausal care and women’s health management.

## 2. Methodology

A comprehensive literature search was conducted to identify relevant studies addressing transdermal hormone therapy in menopause using PubMed, Scopus, Web of Science, and Google Scholar. Publications from 2000 to 2025 were primarily considered, with earlier studies included when relevant to capture both the evolution of the field and the most recent scientific evidence. The search strategy used free-text keywords, including “menopause”, “hormone replacement therapy”, “transdermal hormone therapy”, “estrogen”, “progesterone”, “testosterone”, “hormone delivery systems”, “postmenopause”, and “personalized medicine”. Peer-reviewed original research articles, book chapters, systematic reviews, and meta-analyses involving peri- or postmenopausal women and evaluating transdermal estrogen, progesterone, and/or testosterone therapy were included if they reported clinical outcomes, pharmacokinetic data, safety profiles, or comparative analyses with oral formulations and were published in English. Case reports, editorials, animal or in vitro studies without clinical relevance, studies focusing exclusively on contraceptive hormone use in reproductive-age women, publications lacking sufficient methodological detail or outcome data, and non-English articles were excluded. Titles and abstracts were initially screened for relevance, followed by full-text review for eligibility, and only information directly relevant to the objectives of this review was extracted and synthesized qualitatively. Disagreements regarding study inclusion were resolved through discussion among the authors.

## 3. Endocrine Transitions in Women: From Premenopause to Postmenopause

Studies of female reproductive aging show that hormone changes across menopause follow a predictable biological sequence driven by progressive ovarian follicle loss and secondary neuroendocrine adaptation [9].

During the reproductive years, sufficient ovarian follicles sustain hormone production through coordinated hypothalamic–pituitary–ovarian regulation. Gonadotropin-releasing hormone stimulates pituitary secretion of follicle-stimulating hormone (FSH) and luteinizing hormone (LH), which promote ovulation and the production of estradiol and inhibin B. These ovarian hormones exert negative feedback on the hypothalamus and pituitary, maintaining stable cyclic hormone levels and regular menstruation [9].

Research has shown that the menopausal transition begins when the ovarian follicle pool progressively declines. As follicle number and functional capacity decrease, production of inhibin B and anti-Müllerian hormone falls. Because inhibin B normally restrains FSH secretion, its decline leads to a gradual rise in FSH. At this stage, remaining follicles can still produce estradiol, but hormone output becomes increasingly irregular. Menstrual cycles may persist, yet hormone fluctuations reflect declining ovarian reserve rather than preserved reproductive function [9].

With advancing ovarian aging, follicles become less responsive to pituitary stimulation, and ovulation occurs inconsistently, while age-related hypothalamic changes further disrupt gonadotropin coordination. This combined ovarian and central dysregulation leads to fluctuating estradiol levels, unstable LH surges, increasing anovulatory cycles, and ultimately prolonged amenorrhea culminating in the final menstrual period. Postmenopause represents the endpoint of this process, characterized by the depletion of functional follicles, minimal ovarian hormone production, persistently elevated gonadotropin levels—particularly FSH—and a stable hypoestrogenic state underlying long-term systemic and cutaneous changes [9].

The foundation of female hormonal physiology lies in the coordination of central and peripheral endocrine organs. The hypothalamic–pituitary–ovarian axis, a highly regulated network that includes gonadotropins, ovarian steroids, and dynamic feedback mechanisms, represents the central regulatory pathway of the female endocrine system.

The anterior pituitary releases FSH and LH in response to pulsatile secretion of gonadotropin-releasing hormone by the hypothalamus [10]. A mid-cycle spike in LH triggers ovulation and stimulates the corpus luteum to secrete progesterone during the luteal phase, whereas FSH promotes folliculogenesis and estradiol synthesis in the follicular phase [10,11].

Granulosa cells, which line the ovarian follicles and surround the developing oocyte, produce estrogens—mainly 17β-estradiol—that control menstrual cyclicity, bone mineralization, vascular integrity, neurocognitive function, and secondary sexual characteristics [11,12]. Progesterone, secreted primarily by the corpus luteum formed after ovulation during the luteal phase, regulates the frequency of the gonadotropin-releasing hormone pulse and promotes endometrial receptivity and pregnancy maintenance [11,13]. Both the ovaries and the adrenal glands secrete androgens, such as testosterone and androstenedione. Through classical and paracrine signaling pathways, they play a crucial role in ovarian follicle maturation, sexual desire, and metabolic balance [14,15]. In hyperprolactinemic conditions, anterior pituitary prolactin promotes lactogenesis and inhibits gonadotropin-releasing hormone secretion [16]. Small ovarian follicles secrete anti-Müllerian hormone, an established marker of ovarian reserve and reproductive longevity [17].

Physiological stages and endocrine disorders, including puberty, pregnancy, polycystic ovary syndrome, amenorrhea, and premature ovarian insufficiency, can disrupt hormone balance and lead to menstrual irregularities, infertility, osteoporosis, vasomotor symptoms, mood disturbances, and increased cardiometabolic risk, for which hormone therapy aims to restore endocrine stability [1,18,19]. However, menopause remains the most prominent stage associated with systemic hormone decline and related symptoms, imposing a significant burden on women’s quality of life worldwide. To address these alterations, hormone therapy has long been used to restore endocrine balance and alleviate symptoms, with transdermal formulations emerging as an effective alternative to oral therapy by providing stable hormone levels, reduced hepatic metabolism, and a comparable or lower risk of adverse effects.

The three primary forms of estrogen found in the human body are estrone, 17-β-estradiol, and estriol [20,21]. The strongest naturally occurring estrogen in humans is 17-β-estradiol, which is followed by estrone and estriol. 17-β-Estradiol is the most prevalent estrogen during the premenopausal phase [15,16]. However, during menopause, its levels fall more than those of estrone, making estrone the predominant estrogen during this life stage. A decrease in ovarian follicular activity and a shift in the serum estradiol: estrone ratio are well-established signs of menopause [20,21].

Progesterone is a steroidal hormone essential for regulating the menstrual cycle and supporting the early stages of pregnancy [22]. In women, progesterone is continuously secreted by the corpus luteum throughout the luteal phase of the menstrual cycle, which enhances the secretory activity of the endometrial lining. If fertilization and implantation occur, the presence of human chorionic gonadotropin maintains progesterone production by the corpus luteum [23,24]. Elevated progesterone levels at this stage inhibit menstruation and help establish a uterine environment conducive to supporting embryonic development [23,24]. Conversely, if fertilization does not take place, progesterone levels decline, resulting in decreased endometrial secretion and the onset of menstruation [23].

Fluctuations in progesterone concentrations can contribute to symptoms such as breast tenderness, vaginal dryness, palpitations, and headaches; these symptoms may be alleviated through exogenous progesterone therapy [23,25]. Clinically, progesterone is also employed in the management of menstrual irregularities, abnormal uterine bleeding, menopausal symptoms, and as a component of hormonal contraceptive strategies [23].

During menopause, progesterone levels naturally decline, which contributes to a range of common symptoms such as mood disturbances, hot flashes, vaginal dryness, night sweats, sexual discomfort, headaches, and weight gain [22,26]. As a result, progesterone therapy is often prescribed to alleviate these issues.

In women, testosterone is now acknowledged as a hormone with numerous non-reproductive roles [27]. A deficiency of testosterone is more prevalent in menopausal women than previously recognized, especially among those undergoing hormone replacement therapy or experiencing surgical menopause [27]. While testosterone replacement is a well-established treatment for male hypogonadism—leading to the development of advanced delivery systems designed for physiological hormone release—options for women remain limited, as current methods often result in artificial or supraphysiological androgen levels [27].

Testosterone deficiency in women is increasingly recognized as a clinically significant condition that can affect multiple physiological systems [28]. Although commonly perceived as a male hormone, testosterone plays a vital role in female health, contributing to sexual function, mood regulation, cognitive performance, musculoskeletal integrity, and cardiovascular health [28]. Insufficient testosterone levels have been associated with decreased libido, reduced sexual arousal, and overall diminished sexual satisfaction [29]. Beyond sexual health, testosterone deficiency can manifest as persistent fatigue, low energy, mood disturbances such as depression and anxiety, and cognitive impairments including poor concentration and memory deficits [29].

## 4. Hormonal Management of Menopause: Pharmacologic Agents and Delivery Modalities

**Figure 1:**
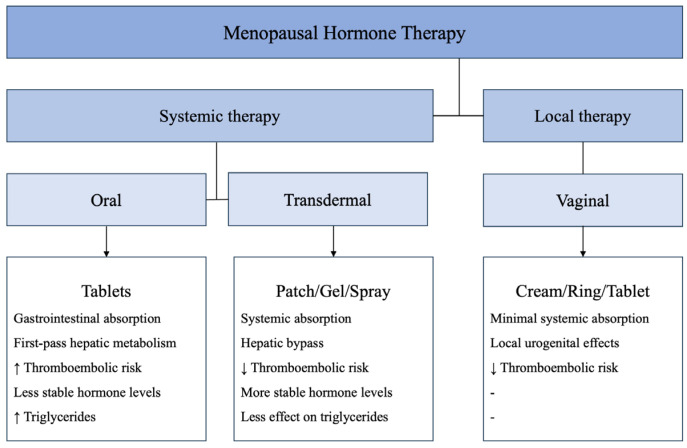
Routes of administration and pharmacokinetic characteristics of menopausal hormone therapy. Systemic therapy includes oral and transdermal formulations, whereas local therapy mainly refers to vaginal administration. Oral formulations are associated with hepatic first-pass metabolism, less stable hormone levels, and may increase thromboembolic risk and triglyceride levels. Transdermal formulations, available as patches, gels, or sprays, bypass first-pass hepatic metabolism, provide more stable hormone levels, and are associated with a lower thromboembolic risk. Vaginal formulations, such as creams, rings, or tablets, exert predominantly local urogenital effects. In the figure, ↑ indicates an increase and ↓ indicates a decrease.

Currently, the Food and Drug Administration has approved hormone therapy for four indications in women going through menopause: moderate to severe vulvovaginal symptoms, prevention of bone loss, vasomotor symptoms, and premature hypoestrogenism [30,31]. Menopausal hormone therapy comes in a variety of forms, dosages, and delivery methods. Since each formulation and delivery method has a different risk–benefit profile and effectiveness, therapy should be tailored to the unique needs and preferences of each patient. Menopausal hormone therapy can be administered systemically via oral and transdermal routes or locally via vaginal formulations. The main routes of administration and their pharmacokinetic characteristics are illustrated in Figure 1. Moreover, regarding dosing, it has been well-established that when prescribing menopausal hormonal therapy, the lowest effective dosage should be used [30,31].

The benefit–risk ratio of menopausal hormone therapy is favorable in women under 60 years of age or within 10 years of menopause onset, particularly for the treatment of vasomotor symptoms and prevention of bone loss, in the absence of contraindications. In contrast, in older women or those further from menopause, the increased risks of dementia, venous thromboembolism, coronary heart disease, and stroke make the benefit–risk profile less favorable [30,31].

Transdermal administration is easy, painless, and helps maintain stable plasma drug concentrations. Another benefit is that, unlike oral formulations, transdermal hormone therapy does not involve the hepatic first-pass metabolism, which reduces the incidence of systemic metabolic side effects and allows the use of reduced doses.

Drug delivery through the skin depends on its anatomy and physiology, with the stratum corneum acting as the main barrier and rate-limiting step. Its variable thickness across different body areas also influences drug permeability. After application, drugs enter the systemic circulation via transcellular, paracellular, and transappendageal pathways. Successful absorption is further determined by the drug’s physicochemical properties, including molecular size, lipophilicity (lipophilic drugs permeate more quickly), melting point, diffusion coefficient, and degree of ionization [21].

Moreover, a known limitation of transdermal drug delivery is the variability in drug absorption both between individuals and within the same person over time. This inconsistency arises from a range of biological factors, including age, sex, ethnicity, skin hydration, underlying health conditions, and the specific site of application on the body [32]. These variables can significantly affect how a drug is absorbed through the skin, posing challenges in achieving predictable therapeutic outcomes [32].

### 4.1. Chemical Characteristics of Estrogens and Progestogens

Estrogens and progestogens used in menopausal hormone therapy differ in their chemical structure, receptor affinity, metabolic pathways, and biological activity. Bioidentical 17β-estradiol exhibits molecular identity to endogenous ovarian estrogen and demonstrates predictable receptor binding and metabolism. In contrast, conjugated equine estrogens represent a heterogeneous mixture of estrogenic compounds with variable pharmacodynamic profiles [21,33].

Similarly, micronized progesterone is structurally identical to endogenous progesterone and is associated with favorable metabolic and cardiovascular profiles, whereas synthetic progestins display diverse chemical modifications that may influence receptor selectivity, lipid metabolism, and thrombotic risk [34,35]. These structural differences contribute to interindividual variability in efficacy and tolerability.

Furthermore, the lipophilic nature of steroid hormones facilitates transdermal absorption, while molecular size and polarity influence skin permeability and systemic bioavailability. Understanding these chemical and pharmacological characteristics is essential for optimizing formulation selection and individualizing therapy [21,36]. To facilitate comparison, the key pharmacological differences between bioidentical and synthetic hormones are summarized in Table 1. These differences are particularly relevant when considering the route of administration, as transdermal delivery may preserve more physiological hormone profiles compared to oral formulations.

In conclusion, as presented in Table 1, bioidentical and synthetic hormones differ in terms of molecular structure, receptor interaction, and metabolic pathways, which may have implications for their pharmacokinetic behavior and clinical use.

### 4.2. Estrogen Delivery Systems

The two indications for estrogen therapy are effective contraception (particularly when paired with progestogens) and the alleviation of menopausal and climacteric symptoms, including vasomotor instability (hot flashes), urogenital atrophy, sleep disturbance, sexual dysfunction, and bone demineralization.

Micronized 17β-estradiol, conjugated equine estrogens, conjugated estrogens, and ethinyl estradiol are among the estrogen formulations available for hormonal therapy. Unlike the synthetic formulations, micronized 17β-estradiol has the same chemical structure as endogenous estradiol and is considered bioidentical [30].

Estrogens are well-absorbed by the gastrointestinal tract, mucous membranes, and skin and are suitable for transdermal administration, while the vehicle’s pH, content, and rate of release from the delivery mechanism all affect how quickly the medication is absorbed [21]. In menopause, estrogen therapy is available in oral, vaginal, and transdermal forms, used alone or in combination with progesterone [36]. In women without a uterus, estrogen alone can generally be administered safely because there is no risk of endometrial cancer [30].

In the United States, oral estrogen therapy continues to represent the predominant form of hormone replacement treatment [21,33]. Increased release of coagulation factors and inflammatory markers, hypertriglyceridemia, an increased risk of venous thromboembolism (VTE), and gallstone formation are among the risks associated with oral estrogens, largely due to hepatic first-pass metabolism [36].

Therefore, as an alternative, the Food and Drug Administration has approved the use of transdermal estrogen formulations that contain plant-derived, bioidentical estradiol, to treat dyspareunia, moderate-to-severe vaginal dryness, and other genitourinary symptoms [21,31]. These preparations employ various delivery systems, including reservoir, matrix, and advanced thermodynamic matrix technologies, and are available as gels, emulsions, sprays, or patches. Most transdermal products deliver estrogen alone, whereas certain delivery-optimized thermodynamic matrix patches also include a progestogen to provide endometrial protection in women with an intact uterus [21,31].

Low-dose vaginal estrogen therapy can be applied as a cream, ring, insert, or tablet, among other forms [36]. Its primary effect is local treatment of postmenopausal vulvovaginal symptoms and, unlike oral formulations, it does not raise systemic estrogen levels above the typical postmenopausal range [36].

When considering that estrogen gels, emulsions, and sprays are all administered directly to the skin, it is expected that surface area, skin thickness, ambient humidity, and application thickness all affect absorption [21,31]. At conventional doses, these formulations typically result in lower serum levels of estradiol compared to patch administration [21,31].

Estrogen gels, emulsions, and sprays usually cause less skin irritation than patches because they do not require adhesives, but future research is necessary to address concerns regarding the transfer of active hormones from one person’s skin to another.

Transdermal gels were first used in Europe in the mid-1970s, and in 2004, the Food and Drug Administration authorized a gel containing 0.06% estradiol. Gels are flammable and should not be exposed to fire or flames until the skin has dried since they depend on the solubility of estrogen in alcohol, which improves the drug’s distribution through the stratum corneum [21].

Emulsions. Advances in nanotechnology have introduced innovative transdermal delivery systems, among which the estradiol emulsion is a notable example. In these formulations, nanoparticles are utilized to optimize the drug’s pharmacokinetic behavior, improving solubility, stability, and systemic absorption [21]. Estradiol emulsions contain micellar nanoparticles. Estradiol, water, oil, alcohol, and surfactant make up the nanoparticles [21]. When estradiol is first applied to the skin, a depot is formed that will eventually diffuse through the stratum corneum and enter the dermal microcirculation [21]. After two weeks of regular use, stable therapeutic levels are reached due to their absorption properties [21].

Estrogen sprays represent a convenient transdermal delivery option, applied directly to the skin through a metered-dose dispenser that releases a standardized amount of hormone with each activation. The spray is applied to a limited surface area and dries quickly, as proven by the absence of transmission to male partners by skin-to-skin contact one hour after application [21,37]. Serum estradiol levels were unaffected by skin washing; however, they did somewhat decrease when sunscreen was applied one hour after the spray [21,37].

Patches. Given that most patches require once or twice every week administration, they offer the advantages of great skin adherence and ease of dosing. Furthermore, a range of doses are accessible in patches, enabling titration [21]. To enable symptom-adapted titration, several patch doses are offered, and while previous reservoir-type patches retain the medication in a reservoir, matrix patches dissolve the drug in liquid or gel. Although skin discomfort may depend on the adhesive properties of a particular patch, matrix patches are less irritating to the skin and therefore better tolerated [21].

Alternatively, estrogen is also used in contraception within modern combined hormonal formulations that pair a progestogen with one of four estrogens: ethinyl estradiol (EE), 17β-estradiol (E2), estradiol valerate (E2V; a prodrug of E2), or estetrol (E4) [38]. However, oral formulations predominate in this category, whereas the available transdermal patches and vaginal rings are ethinyl estradiol-based [38,39]. Combined oral contraceptives typically contain 20–35 µg ethinyl estradiol per tablet. Dosing of less than 35 µg ethinyl estradiol is standard to mitigate the thromboembolic and metabolic side effects [26]. Different preparations and regimens for contraception should be tailored to each patient’s preferences, lifestyle, medical history, and risk profile [38].

In conclusion, transdermal estrogen therapy in menopause is well-tolerated, maintains stable plasma concentrations, reduces systemic metabolic side effects, and allows lower dosing. Compared with oral formulations, it may also reduce the risk of stroke and venous thromboembolism, supporting its role as a preferred first-line option [40,41,42,43,44,45]. Different transdermal formulations present distinct characteristics: patches provide continuous and consistent delivery but may cause local irritation; gels and emulsions allow flexible dosing with generally better skin tolerability, though absorption may vary; and sprays offer convenient application but limited dosing flexibility. Therefore, the choice of formulation should be individualized based on patient preference, skin sensitivity, dosing needs, and lifestyle factors.

### 4.3. Progesterone Delivery Systems

There are many different progestogens accessible globally, either as mono-substances for free combinations with estrogens or as fixed combinations [34]. Progesterone, 19-nortestosterone, and spironolactone derivatives are the three synthetic progestins that are available, along with natural micronized progesterone [34].

Progesterone can be delivered using multiple administration routes, such as intravenous, subcutaneous, intramuscular, rectal, vaginal, transdermal, intranasal, and oral methods [22]. The choice of delivery pathway is critical, as it significantly influences the hormone’s pharmacokinetic profile and overall therapeutic effectiveness [22]. For instance, oral progesterone exhibits notably low bioavailability, typically under 10%, primarily due to limited absorption in the gastrointestinal tract and substantial metabolism by the liver during first-pass clearance [22,46,47]. The vaginal route is often favored due to its ability to deliver localized effects, user convenience, rapid absorption, and enhanced bioavailability. However, it may be associated with adverse effects such as irritation and occasional bleeding [48,49].

Many of the limitations associated with conventional progesterone delivery can be addressed through the development of advanced drug delivery technologies, such as hydrogels, lipid-based systems, various nanocarrier platforms, polymeric carriers, and controlled or depot-release formulations [23]. Among these, transdermal natural progesterone creams have gained considerable attention in both popular media and among women, emerging as a potential alternative to traditional hormone replacement therapies [35]. However, based on the current body of evidence, the use of progesterone cream in postmenopausal treatment remains unproven. Its application should be confined to rigorously designed clinical trials aimed at evaluating its safety and therapeutic effectiveness [35].

Various transdermal delivery systems have been designed to provide consistent drug release through intact skin and, similarly to estrogen therapy, the stratum corneum, the skin’s outermost layer acts as both a barrier and a drug reservoir, influencing absorption rates [50,51]. Although progesterone is capable of permeating the skin, it is rapidly metabolized by local enzymes such as 5α-reductase, limiting its systemic bioavailability [50,51]. However, when applied topically to the breast, significant local tissue concentrations can be achieved, suggesting a potential utility for site-specific therapy [42].

Clinical inconsistency exists in studies examining serum progesterone levels after topical or transdermal application, with some showing minimal changes despite observable effects on target tissues. While topical progesterone can lead to elevated levels in saliva and potentially other tissues, serum levels may remain low, leading to confusion about its effectiveness. Studies have also demonstrated significant increases in salivary progesterone and tissue concentrations (such as in the breast), serum levels, however, may not reflect these increases [52,53]. Progesterone, being lipophilic, may preferentially accumulate in fatty tissues after absorption through the skin, rather than circulating in the bloodstream in high concentrations [52,54]. Red blood cells may act as a reservoir for progesterone, carrying it to target tissues, including salivary glands, while serum levels remain relatively low [52]. One study suggested that capillary blood, closer to the site of absorption, may show higher progesterone levels than venous blood after topical application [53]. This inconsistency suggests that relying solely on serum progesterone levels to assess the effectiveness of topical progesterone therapy may be misleading [53]. Further research is needed to fully understand the mechanisms of progesterone absorption, distribution, and metabolism after topical application, and to determine the most appropriate methods for monitoring its effects [52,55].

Among the strategies developed to enhance drug permeation through the skin, microemulsions have gained attention as a versatile and promising delivery platform [56]. These formulations are thermodynamically stable, easy to prepare, and capable of incorporating both water- and lipid-soluble drugs [56].

Recent advances in nanotechnology have led to the development of smart transdermal systems such as nanostructured lipid carriers (NLCs), solid lipid nanoparticles (SLNs), and nanogels, which enhance skin penetration, improve drug stability, and offer sustained release profiles. However, these approaches are not yet widely available in clinical practice, with only limited examples currently available and therefore remain outside the scope of routine therapeutic use. As increasing data are being generated, a brief overview may help familiarize readers with these evolving concepts.

Nanostructured lipid carriers are a promising drug delivery system for progesterone and offer several advantages over traditional formulations, including improved drug loading, controlled release, and enhanced skin penetration [57,58,59]. NLCs are colloidal particles composed of a mixture of solid and liquid lipids, a structure that allows for higher drug encapsulation and tunable drug release profiles [60]. NLCs can facilitate the penetration of progesterone through the skin, potentially improving its bioavailability and therapeutic effect [58].

Solid lipid nanoparticles are emerging as a promising vehicle for transdermal drug delivery and are currently under investigation for the administration of progesterone. These lipid-based nanocarriers offer several advantages, including improved drug solubility and stability, controlled release profiles, and a potential reduction in systemic side effects [61,62]. For progesterone, which suffers from poor oral bioavailability due to extensive hepatic first-pass metabolism, SLNs represent an innovative approach to enhance therapeutic efficacy via transdermal application [63].

As a lipophilic molecule, progesterone demonstrates limited aqueous solubility, which can hinder absorption through conventional delivery routes, while encapsulation through biocompatible lipid matrices enhances absorption through the skin [63,64]. Localized delivery through the skin enables targeted therapeutic action while limiting systemic hormone exposure [65].

Nanogels, which are nanoscale hydrogels, are being explored as another promising drug delivery system for progesterone [66]. They offer several advantages, including high drug loading capacity, biocompatibility, and the ability to control drug release [67]. The amphiphilic nature of nanogels allows for the incorporation of hydrophobic drugs like progesterone, potentially improving their bioavailability and therapeutic effect [66].

Transfersomes, a type of ultradeformable vesicle, are being used for the transdermal delivery of progesterone, improving its bioavailability and therapeutic efficacy [68]. These vesicles, composed of lipids and a surfactant, can penetrate the skin’s stratum corneum, enhancing drug penetration and potentially offering a more effective route for progesterone delivery [68].

Emerging materials like bioresponsive polymers hold significant promise for advancing transdermal progesterone delivery by enabling controlled and targeted release based on specific biological stimuli [69]. These polymers can be designed to respond to changes in pH, temperature, or enzyme activity within the skin, leading to a more efficient and personalized delivery system [69,70]. By incorporating bioresponsive polymers into transdermal patches, progesterone delivery can be modulated based on the local environment of the skin and therefore release more progesterone in an acidic environment, which can occur in certain skin conditions [69,70,71].

### 4.4. Testosterone Delivery Systems

Testosterone delivery presents multiple clinical challenges, particularly in achieving stable hormone levels, minimizing adverse effects, and ensuring long-term patient compliance [72].

The pharmacokinetics of testosterone are heavily influenced by the chosen delivery route, with oral administration being largely ineffective due to extensive hepatic first-pass metabolism, resulting in insufficient systemic bioavailability [72,73]. Even with alternative formulations, such as intramuscular injections, transdermal patches, and topical gels, variability in absorption and metabolism often results in fluctuating serum testosterone levels, compromising symptom control and increasing the risk of adverse effects [72,74]. After transdermal absorption, testosterone binds primarily to sex hormone-binding globulin (SHBG) and albumin. Interaction with SHBG is crucial, as changes in SHBG levels can significantly impact free testosterone availability [75]. Moreover, testosterone is metabolized in the skin and systemically to dihydrotestosterone (DHT) and to estradiol in various tissues [76]. Individual patient factors, including body composition, metabolic rate, and comorbidities, further complicate the optimization of dosing and delivery strategies [72].

Adverse effects also pose a significant limitation to testosterone therapy. Intramuscular injections may cause pain, bruising, or pronounced hormonal fluctuations, which can affect mood, libido, and energy levels [77]. Transdermal systems are associated with local skin reactions, including irritation and erythema, while topical gels carry the risk of accidental transfer through direct skin contact, potentially exposing partners or children to unintended androgenic effects [74].

Use of testosterone therapy in women is primarily considered for specific clinical conditions where testosterone deficiency is implicated and other causes have been excluded [78]. It is crucial to note that, as of the current date, no transdermal testosterone product is universally approved by major regulatory bodies (e.g., FDA in the United States) specifically for use in women, leading to off-label use of male formulations or compounded preparations [79,80].

Primary indications of testosterone therapy include:

#### 4.4.1. Hypoactive Sexual Desire Disorder (HSDD)

HSDD, characterized by a persistent or recurrent deficiency or absence of sexual desire for sexual activity that causes marked distress or interpersonal difficulty, is the most extensively studied indication for testosterone therapy in women [80,81]. Clinical trials have demonstrated that transdermal testosterone can significantly improve sexual desire, arousal, and orgasm frequency [80,82].

#### 4.4.2. Surgical Menopause (Bilateral Oophorectomy)

Women undergoing bilateral oophorectomy experience an abrupt and significant decline in endogenous testosterone production, often leading to a more pronounced and rapid onset of androgen deficiency symptoms compared to natural menopause [78,83,84]. Testosterone replacement therapy, including transdermal options, may be considered in these women to alleviate symptoms [78].

#### 4.4.3. Adrenal Insufficiency

In women with primary adrenal insufficiency, endogenous adrenal androgen production is severely compromised; some women may benefit from low-dose androgen replacement, including testosterone [85].

The major challenge in transdermal testosterone therapy for women is the absence of widely available, specifically formulated, low-dose products. Current clinical practice heavily relies on the strategic use of existing male formulations or custom-compounded preparations [80,86].

Hydroalcoholic gels (e.g., AndroGel^®^, Testim^®^) typically consist of testosterone dissolved in an alcohol-based solvent (e.g., ethanol, isopropanol) with gelling agents (e.g., carbomers) [87,88,89]. The high alcohol content facilitates rapid evaporation upon skin application, leading to a supersaturated state of testosterone on the skin surface [90], enhancing the partitioning of testosterone into the stratum corneum [91]. The alcohol also acts as a permeation enhancer, temporarily disrupting the lipid bilayers of the stratum corneum [92].

Emollient-based creams and gels (often compounded) incorporate testosterone into an oil-in-water or water-in-oil emulsion base and may offer a more sustained release profile and potentially less skin irritation due to the emollient properties [93].

Transdermal patches are designed to deliver testosterone at a relatively constant rate over a prolonged period [94]. Permeation enhancers are often incorporated into the adhesive or matrix to improve drug flux across the skin [94,95]. Male testosterone patches (e.g., Androderm^®^) are generally too high-dose for women, making their off-label use complex and risky due to the challenges of dose reduction [96]. A testosterone patch (Intrinsa/Esstara) was developed for HSDD in postmenopausal women but was not approved worldwide due to concerns about long-term safety data [80,82,97].

### 4.5. Combined Menopausal Hormonal Therapy with Estrogens and Progestogens

The combination of estrogen with a progestogen represents the cornerstone of menopausal hormone therapy for women with an intact uterus. The addition of a progestogen provides endometrial protection, allowing safe long-term use of estrogen therapy [31]. Additionally, compared to non-menopause hormonal therapy use, a recent meta-analysis found that continuous combined menopause hormonal therapy significantly decreased the incidence of endometrial cancer [34,98]. The authors did not, however, distinguish between progestogen types and hormonal therapy regimens [34,98]. Endometrial biopsies are recommended before treatment initiation and at 12 months to demonstrate endometrial safety for regulatory approval of certain fixed combined menopausal hormone therapies. Strict regulations apply regarding histopathological evaluation and clinical correlation to ensure appropriate implementation [99].

However, there are also downsides of combined menopausal hormone therapy, as the addition of progestogens may lead to adverse effects such as irritability and menstrual bleeding [100]. The incidence of adverse reactions may be reduced using an intrauterine administration of progestogens.

Over time, various formulations and delivery systems, including oral, transdermal, and intrauterine options, have been developed to optimize efficacy, safety, and patient adherence. Treatment may be administered cyclically, with the progestogen administered 12–14 days per month, or continuously, with both hormones taken daily [30].

Levonorgestrel, a second-generation synthetic progestogen, is primarily approved as a first-line oral emergency contraceptive. It is also available in various formulations, including transdermal systems, oral combination pills with estradiol, and intrauterine devices, the latter providing up to five years of contraception. In addition to its contraceptive role, levonorgestrel is used off-label for conditions such as endometrial hyperplasia, endometriosis, menorrhagia, and in menopausal hormone therapy [101]. A randomized trial showed that intrauterine levonorgestrel combined with transdermal estrogen is as effective as oral estrogen–progestogen therapy in relieving climacteric symptoms. Although early spotting was more frequent, bleeding patterns became similar over time, supporting it as a safe and effective alternative for progestogen administration in menopausal hormone therapy [100].

Moreover, in a more recent systematic review [34], the safety of intrauterine levonorgestrel in continuous combined menopausal hormonal therapy was evaluated through five randomized controlled trials that included perimenopausal and/or postmenopausal subjects, with doses ranging from 5 to 20 mcg/day, associated with oral and transdermal estradiol valerate, with no reports of endometrial hyperplasia or malignancy [100,102,103,104,105].

Norethisterone acetate is a synthetic progestogen widely used in gynecological practice to regulate menstrual disorders and manage various hormone-related conditions.

In addition to its established gynecological uses, norethisterone acetate has been investigated in the context of menopause-related cardiovascular risk, which is increased due to metabolic and clinical changes such as insulin resistance, dyslipidemia, and hypertension [106]. Furthermore, when combined with transdermal estrogen, it has shown favorable effects on lipid profile and coagulation parameters, suggesting a potential role in reducing cardiovascular risk [107].

## 5. Effects of Transdermal Versus Oral Hormone Replacement Therapy in Postmenopause

Hormone replacement therapy (HRT) is fundamental in the management of menopausal symptoms and the prevention of long-term estrogen deficiency–related conditions such as osteoporosis. As previously stated, among the various administration routes that are currently available, oral and transdermal delivery are the most commonly used. While oral HRT has historically been the standard approach, concerns regarding hepatic first-pass metabolism and its association with an increased risk of thromboembolic events and alterations in lipid profiles have prompted the exploration of alternative delivery systems [108].

The key pharmacokinetic differences between different delivery options of menopausal hormone therapy, including the role of hepatic first-pass metabolism and their clinical implications, are illustrated in Figure 1.

Firstly, while adverse reactions are well-recognized risks associated with oral administration, there has been increasing interest in whether switching to the transdermal route can provide comparable efficacy.

Recent studies have shown that both oral and transdermal estrogen formulations demonstrate comparable efficacy in improving menopausal symptoms and overall quality of life in perimenopausal and recently postmenopausal women [109,110]. Findings from randomized controlled trials, including a study involving 257 women assessed with the Menopause-Specific Quality of Life (MENQOL) questionnaire, indicated significant symptom improvement with both routes of administration. While overall treatment outcomes were similar, oral estrogen showed a slightly greater effect on vasomotor symptoms at 24 weeks [109,110]. These results support the use of either formulation in clinical practice, allowing for individualized therapy based on patient preference, tolerability, and risk profile [109,110].

With regard to menopausal vasomotor symptoms, transdermal hormone replacement therapy is widely used. In comparing the efficacy and local tolerability of a transdermal patch versus a metered-dose spray, a network meta-analysis of 46 studies showed that both delivery methods significantly reduced hot flashes compared to the placebo, with no notable difference in efficacy between them, but better local skin tolerability with the metered-dose spray than the patch [111].

Moreover, transdermal hormone therapy demonstrated superior efficacy in improving sleep quality compared to oral hormone therapy, according to evidence from a pooled analysis of 15 randomized controlled trials encompassing 27,715 participants [112]. These trials investigated the impact of different hormone therapy regimens on subjective sleep disturbances in menopausal women [112]. The analysis revealed that transdermal administration of hormones, particularly 17β-estradiol and conjugated equine estrogens, was more beneficial in enhancing sleep quality, whereas oral formulations, including estradiol valerate, showed less consistent or limited effects [112]. These findings support the clinical preference for transdermal routes in managing sleep-related symptoms during menopause [112].

In evaluating the effectiveness of transdermal hormone replacement therapy, its impact on lipid metabolism is an important consideration. Findings from a randomized, double-blind, placebo-controlled trial conducted in 2021 provide detailed insights into these effects [113]. Transdermal estrogen does not significantly alter HDL-C concentrations but reduces LDL-C and total cholesterol, supporting its beneficial impact on cardiovascular risk markers [113]. Although HDL-C levels remain unchanged, transdermal estrogen modifies HDL functionality by decreasing HDL cholesterol efflux capacity, particularly ABCA1-specific cholesterol efflux capacity, and influences HDL particle composition [113]. Specifically, it promotes a shift toward larger HDL particles while reducing the proportion of small HDL particles, changes that may have clinical relevance for cardiovascular health. Total HDL particle concentration and triglyceride levels remain unaffected. Additionally, transdermal administration avoids hepatic first-pass metabolism, which may explain the absence of adverse effects on lipid profiles commonly seen with oral estrogen [113]. While the clinical implications of these detailed changes in HDL function and composition remain to be fully established, the overall lipid profile modifications observed with transdermal HRT suggest a beneficial role in cardiovascular risk management [113].

Reduced sexual desire is a common concern in postmenopausal women, affecting approximately 40% of women and often leading to significant emotional and relationship distress. Although estrogen therapy effectively addresses vaginal atrophy and associated discomfort, it does not improve sexual desire. Testosterone has been increasingly recognized for its role in female sexual function, and several randomized controlled trials have demonstrated that testosterone therapy, especially when delivered transdermally, can lead to significant improvements in sexual desire and the frequency of satisfying sexual experiences, with only mild adverse effects reported [114]. Earlier studies using oral or intramuscular testosterone showed some benefit, but frequently resulted in supraphysiological hormone levels. Oral formulations, in particular, were linked to unfavorable lipid profile changes due to hepatic first-pass metabolism [115,116]. In contrast, transdermal administration, through patches, gels, or creams, bypasses the liver, thereby avoiding these metabolic side effects while maintaining stable serum testosterone levels similar to those seen in premenopausal women. This route has become the preferred method in clinical studies evaluating testosterone therapy in women [115,116]. Despite the growing evidence supporting its efficacy and safety, there is currently no testosterone product specifically approved for women in the European market, and therefore, treatment often involves adapting male-formulated testosterone preparations, which contain higher hormone concentrations, creating challenges for appropriate dosing [114].

In clinical practice, the choice between oral and transdermal menopausal hormone therapy is guided by individual risk profiles, based on the most important characteristics of each product that might influence the decision, as summarized in Table 2, which outlines the key pharmacokinetic and clinical differences between oral and transdermal HRT based on available evidence [108,109,110,111,112,113]. These differences are largely explained by the avoidance of hepatic first-pass metabolism in transdermal therapy, which results in more stable hormone levels and a reduced impact on coagulation and lipid metabolism compared to oral formulations. Oral formulations may be considered in younger, low-risk women without cardiovascular, thromboembolic, or metabolic risk factors.

In contrast, transdermal therapy is generally preferred in women with obesity, hypertension, diabetes, migraine, smoking history, hepatic dysfunction, or increased risk of venous thromboembolism, due to the avoidance of hepatic first-pass metabolism and more stable pharmacokinetic profile.

### Personalized Approaches to Menopausal Hormone Therapy

Personalized medicine in menopausal hormone therapy is based on the integration of multiple patient-specific factors, including age, time since menopause, cardiovascular and thromboembolic risk profile, metabolic status, comorbidities, and individual symptom burden [30,31]. Clinical practice guidelines emphasize individualized decision-making, particularly with regard to the timing of initiation and baseline risk assessment.

The “timing hypothesis” suggests that initiation of hormone therapy within 10 years of menopause onset or before the age of 60 is associated with a more favorable benefit–risk ratio compared with later initiation. This concept represents a central element of personalized menopausal care and supports early, carefully selected treatment in symptomatic women without major contraindications. Personalization also involves the selection of the appropriate molecule and route of administration. Bioidentical 17β-estradiol differs pharmacodynamically and metabolically from conjugated equine estrogens, while micronized progesterone exhibits a different safety and tolerability profile compared with synthetic progestins [34,35]. In women with increased thrombotic or metabolic risk, transdermal delivery is often preferred due to the avoidance of hepatic first-pass metabolism and the provision of more stable serum hormone concentrations [21,33,36,41,44].

Dose individualization represents another essential component of personalized therapy. Treatment should be tailored to symptom severity and regularly reassessed to minimize long-term exposure and adverse effects [30,31]. Emerging insights in pharmacogenetics and pharmacogenomics further support the concept of individualized hormone therapy, as genetic variability may influence hormone metabolism, receptor sensitivity, and susceptibility to adverse events [80,86]. Although clinical application of pharmacogenomic testing remains limited, future research may refine patient selection and dosing strategies.

Overall, personalized menopausal hormone therapy represents a dynamic, patient-centered approach that integrates the timing of initiation, molecular selection, route of administration, dosing strategy, and individual risk stratification to optimize therapeutic outcomes.

## 6. Effects and Clinical Outcomes of Menopausal Hormone Replacement Therapy

### 6.1. Aging and Skin Rejuvenation

Menopausal hormone therapy enhances dermal elasticity and collagen deposition, leading to diminished wrinkle severity and greater cutaneous thickness. However, additional rigorously designed clinical studies are necessary to address the remaining uncertainties in an evidence-based framework [117].

Facial aging is predominantly influenced by genetic predisposition, environmental exposures, diet, and additional factors. Ultraviolet radiation accelerates these processes, and photoprotection remains the sole validated strategy to delay them. Cigarette smoke exerts cytotoxic effects and should be avoided to prevent premature cutaneous aging. Structural and functional alterations of senescent skin represent a physiological phenomenon but may predispose individuals to clinically significant dermatologic complications. An increasing proportion of older individuals are dissatisfied with visible age-related changes and seek therapeutic options. Although substantial benefits can be obtained through current anti-aging modalities, none provide complete reversal [117,118].

Cutaneous status and menopause are strongly interconnected. Across all life stages, women aspire to maintain healthy skin. Age-related dermal decline arises primarily from the accumulation of reactive oxygen species generated during cellular metabolism. Additionally, reduced concentrations of sex steroids contribute to delayed epidermal renewal, linked at the molecular level to protein imbalance, dysregulated cytokine signaling, and persistent inflammation. To address these mechanisms, dermatologic and cosmeceutical treatments have been developed. Effective therapies may enhance dermal density, thickness, hydration, and firmness while simultaneously reducing wrinkle formation [117,119,120].

Estrogen receptors are expressed in multiple cutaneous structures, such as keratinocytes, melanocytes, fibroblasts, pilosebaceous units, and sebaceous glands; therefore, estrogen deficiency during menopause significantly influences skin physiology. Evidence indicates that postmenopausal skin exhibits thinning and reduced elasticity, and the administration of estrogen has been associated with improvements in surface morphology, hydration capacity, dermal collagen levels, and biomechanical resilience [117,121].

Therefore, recent efforts have been made to identify the optimal formula of hormonal therapy for restoring estrogen and progesterone concentrations to levels similar to those observed in the premenopausal state that may help re-establish their physiological benefits on skin structure and function. Although transdermal hormone therapy demonstrates favorable safety outcomes compared with oral administration, particularly in relation to thromboembolic risk and effects on adipose tissue distribution, current evidence regarding its comparative efficacy, optimal dosing, and formulation for dermatologic outcomes remains limited, highlighting the need for further clinical investigation [117,121,122,123,124,125].

### 6.2. Memory

Older women appear to exhibit a greater susceptibility to cognitive decline compared with men. The potential role of menopausal hormone therapy (MHT) in enhancing memory among postmenopausal women remains uncertain [126].

Alzheimer’s disease (AD) represents the leading etiology of dementia, predominantly affecting older individuals, and typically presents first with memory impairment. Cognitive function encompasses multiple domains ranging from memory to executive abilities, with memory dysfunction, particularly verbal memory, representing the most frequent early symptom and strongest predictor of Alzheimer’s disease. Epidemiological data indicate a greater frequency of AD in aging women compared with men, potentially linked to reduced estrogen concentrations. Experimental models and in vitro research have demonstrated neuroprotective actions of estrogen on cerebral structures, particularly those involved in cognitive function [126,127,128,129,130].

In contrast, emerging experimental and observational studies suggest potential neuroprotective mechanisms of estrogen; however, these hypotheses remain incompletely validated in large clinical trials.

Certain epidemiological investigations indicate that MHT might confer protection against cognitive deterioration and reduce the risk of dementia in postmenopausal women [126,131,132,133]. In contrast, the Nurses’ Health Study, which monitored 13,807 women aged 70–81 years, reported that prolonged hormone use (≥5–10 years) was associated with a higher likelihood of global cognitive impairment and deficits in verbal memory. Overall, findings regarding the influence of MHT on memory—particularly verbal domains—remain inconsistent. While some studies demonstrated enhanced verbal memory performance in women receiving MHT, several large-scale trials found no measurable benefit of hormone therapy on this function [126,134,135,136,137].

The WHI Study of Cognitive Aging (WHISCA) reported that combined estrogen–progestin therapy adversely affected verbal memory in postmenopausal women. To explain the conflicting findings regarding estrogen and cognition, the “critical window” hypothesis was introduced. This concept suggests that the cognitive influence of MHT depends on the timing of initiation. When administered during early menopause, hormone treatment may attenuate cognitive decline, whereas delayed initiation, several years after menopause, appears ineffective or potentially detrimental [126,138,139,140].

The meta-analysis by Chen L et al. demonstrated no significant differences between hormone therapy and the placebo in logical memory among postmenopausal participants, including those in early menopause [126]. Furthermore, MHT exhibited neither beneficial nor detrimental effects on delayed recall assessed by the California Verbal Learning Test or on backward and total digit span, though a potential negative impact on forward digit span was observed [126]. These findings indicate that MHT does not improve verbal memory and may slightly impair short-term memory [126]. Overall, the current evidence from randomized controlled trials and meta-analyses does not support a significant beneficial effect of menopausal hormone therapy on verbal memory or global cognitive performance.

In summary, hormone therapy does not appear to improve verbal memory in postmenopausal women and may negatively affect certain aspects of short-term memory. Current evidence does not justify prescribing menopausal hormone therapy for cognitive improvement in women under 60, including those recently entering menopause. Nevertheless, additional large, high-quality randomized controlled trials are required to clarify the influence of MHT on memory. Future investigations should prioritize recently menopausal populations, direct comparisons between different estrogen and progestin formulations, and the impact of treatment duration [126].

### 6.3. Risk of Melanoma and Keratinocyte Skin Cancer

The following subsection addresses potential safety considerations related to transdermal hormone therapy, focusing on possible associations and general risk trends rather than on directly attributable adverse reactions. In recent years, an increasing proportion of women manage menopausal symptoms through hormonal interventions, which have demonstrated an improved safety profile. Nevertheless, the potential link between MHT and oncogenesis remains a significant concern, particularly regarding breast and endometrial malignancies [141,142,143].

The incidence of cutaneous cancers, including melanoma and keratinocyte carcinomas, has markedly risen in recent decades. Beyond established risk determinants and the pathogenic role of ultraviolet radiation, accumulating evidence suggests a hormonal influence on skin tumorigenesis [141,143,144,145]. Estrogens exert multiple regulatory effects on cutaneous physiology, including the modulation of dermal thickness and facilitation of wound repair. Moreover, they are recognized as major contributors to sex-based differences in melanoma incidence and outcomes. Although melanoma occurs more frequently in men overall, women predominate in the 20–45-year age group. Additionally, female patients generally experience a more favorable prognosis; however, this survival advantage diminishes after menopause, coinciding with declining estrogen concentrations [141,146,147,148,149,150].

Further evidence supporting the influence of sex hormones on cutaneous tumor biology arises from observed changes in nevi and melanoma during pregnancy. Nevi have been described as increasing in size and pigmentation, possibly due to estrogen receptor activation in melanocytes. Moreover, melanoma represents the most frequently diagnosed malignancy during gestation, and a recent meta-analysis demonstrated a poorer prognosis for pregnancy-associated cases. Research exploring reproductive variables has also indicated that prolonged estrogen exposure, such as through multiparity or early onset of menarche, may modulate melanoma susceptibility [141,151,152,153,154,155].

Regarding keratinocyte carcinoma development, estrogens have been proposed to function as mitogenic stimuli for keratinocytes and/or as agents that enhance photosensitivity. Since keratinocyte carcinoma predominantly affects older populations, who often receive menopausal hormone therapy, elucidating the potential contribution of hormonal treatment to basal cell carcinoma and squamous cell carcinoma pathogenesis remains a critical area of investigation [141,156].

The administration of menopausal hormone therapy may elevate melanoma risk among postmenopausal women, particularly influencing specific histopathological variants such as superficial spreading melanoma and lentigo maligna melanoma [141]. Both estrogen-only therapy and combined estrogen–progestogen therapy were associated with a significant increase in risk, predominantly observed in current users [141]. Moreover, MHT exposure was linked to a higher incidence of keratinocyte carcinomas, primarily related to estrogen-only therapy administration, the basal cell carcinoma subtype, ongoing therapy, and prolonged treatment duration exceeding five years [141].

From a mechanistic standpoint, estrogens function as proliferative mediators in both melanocytes and melanoma cells, exerting biological influence via genomic mechanisms mediated by nuclear estrogen receptors (ERs) and non-genomic signaling pathways. Comparable to other hormone-responsive malignancies, such as breast and prostate carcinoma, two primary ER subtypes have been identified: ERα, which predominates in the epidermis and is associated with proliferative activity and tumorigenic potential, and ERβ, the principal receptor expressed in melanocytes. The expression of ERβ is inversely related to Breslow depth and melanoma progression, conferring antineoplastic effects. As various ligands demonstrate distinct binding affinities for ERs, maintaining equilibrium in the ERα/ERβ ratio remains crucial for cellular homeostasis. Furthermore, the G protein-coupled estrogen receptor, acting through non-genomic pathways, synergizes with ERβ, resulting in thinner tumor lesions and more favorable melanoma outcomes [141,157,158,159].

Estrogens also possess photosensitizing properties, altering cutaneous susceptibility to UV radiation. This is clinically significant, as UV-induced skin injury represents a major etiological factor in keratinocyte carcinoma development, promoting mutational accumulation in keratinocytes, defective DNA repair processes, and attenuated immune surveillance within the tumor microenvironment. Additionally, estrogens stimulate keratinocyte proliferation. Consequently, estrogenic activity appears dual in nature, promoting carcinogenesis on the one hand and enhancing the proliferation of genetically altered cells on the other [141,160].

In conclusion, MHT has been linked to a higher incidence of melanoma and non-melanoma skin cancers. Variables including formulation type, tumor histopathology, and treatment duration may influence this association. Regular dermatologic monitoring is advisable for women undergoing MHT [141].

### 6.4. Preventing Cardiovascular Disease

Findings from systematic reviews of observational research indicate that menopausal hormone therapy may lower the occurrence of cardiovascular events in postmenopausal women; however, outcomes from randomized controlled trials remain inconsistent [161].

Due to reduced estrogen concentrations and the consequent loss of its cardioprotective effects, older women, particularly those who are postmenopausal, exhibit an elevated susceptibility to cardiovascular disease. Hormone replacement therapy is commonly administered in this population to alleviate menopausal symptoms, including vasomotor and urogenital disturbances. During this stage, several risk factors, such as diabetes mellitus, dyslipidemia, and hypertension, become more prevalent. Women generally develop cardiovascular disease later than men; however, the perimenopausal period is characterized by heightened cardiovascular risk, rendering postmenopausal women a high-risk group. This vulnerability has been linked to physiological changes associated with menopause, as evidenced in longitudinal studies, including alterations in sex hormone levels, serum lipids, lipoproteins, and redistribution of body fat. Collectively, these observations underscore the importance of careful monitoring of cardiovascular risk factors in postmenopausal women [162,163,164,165,166,167].

Estrogen is a key modulator in lowering cardiovascular disease risk, and its decline following menopause may justify the use of hormone replacement therapy to mitigate this risk. For example, transdermal estrogen formulations have been shown to confer cardiovascular protective effects [168,169,170].

Administration of transdermal 17β-estradiol combined with norethisterone acetate favorably influenced several cardiovascular risk markers in postmenopausal women, as demonstrated by significant reductions in fibrinogen, factor VII, LDL cholesterol, and total cholesterol. Given the central role of these parameters in cardiovascular protection, transdermal 17β-estradiol plus norethisterone acetate may represent a therapeutic option for postmenopausal women at elevated cardiovascular risk [107].

However, in the overall study population, there is no indication that hormone therapy contributes to the prevention or management of cardiovascular disease. No robust evidence demonstrated an effect of hormone therapy on all-cause mortality, cardiovascular death, non-fatal myocardial infarction, angina, or revascularization rates. Conversely, therapy was associated with elevated risks of stroke, venous thromboembolism, and pulmonary embolism. Year-by-year analyses of mortality revealed no significant differences between intervention and control groups. Similarly, survival did not differ substantially with cumulative treatment duration, except after ten years of therapy, when a modest survival advantage was observed in the hormone therapy cohort [161].

### 6.5. Drug Interactions

Drug interactions may influence not only the safety but also the effectiveness of menopausal hormone therapy, particularly in patients receiving multiple concomitant medications, thereby representing an important determinant of clinical outcomes. This subsection discusses clinically relevant safety considerations and potential drug interactions, emphasizing broader clinical implications rather than direct adverse effects attributable to hormone therapy. Menopausal hormone therapy is commonly prescribed, with guidance provided by multiple international professional organizations; nevertheless, a comprehensive evaluation of potential pharmacological interactions with MHT is lacking, despite the fact that many women in midlife concurrently use medications for other health conditions [171].

A systematic review by Fasero et al. categorized 23 pharmacological groups based on their interaction potential with MHT, highlighting that while vaginal MHT formulations generally pose minimal risk, oral and transdermal MHT may interact with various drug classes, necessitating careful evaluation [171].

Clinically significant interactions include the effect of MHT on thyroid hormone replacement therapy. Oral estrogen therapies can increase thyroxine-binding globulin levels, potentially reducing the bioavailability of free thyroid hormones and necessitating adjustments in thyroid hormone dosages [172].

Additionally, MHT can influence coagulation parameters, affecting the metabolism of other medications. For instance, oral estrogens may interact with drugs metabolized by the cytochrome P450 enzyme system, such as carbamazepine and rifampin, potentially altering their efficacy [173].

Current evidence regarding interactions between hormone therapy and other medications is limited and predominantly indirect. Available data are derived from biological plausibility, extensive concomitant use without reported adverse events, or extrapolation from studies on hormonal contraception, yet several pharmacological classes provide information that can guide clinical practice. Clinical decisions should follow these guidelines while also considering individualized risk–benefit assessments, taking into account the patient’s underlying conditions, specific therapeutic needs, the availability of alternative treatments, and the level of evidence that supports current findings in order to assure clinical relevance in each patient setting, as summarized in Table 3, which outlines the main clinically relevant drug interactions, their mechanisms, and their implications based on available evidence [171,172,173]. Overall, clinically significant interactions are mainly associated with oral estrogen therapy due to hepatic first-pass metabolism, whereas transdermal formulations appear to have a lower interaction potential.

## 7. Safety Profile, Adverse Effects, Events, and Complications

While the previous section primarily focused on therapeutic efficacy and the broader clinical effects of transdermal menopausal hormone therapy, it also includes discussions of general associations and long-term considerations, such as cancer risk and skin aging, which are not classified as direct adverse reactions but rather as clinically relevant outcomes reported in the literature. Therefore, the following section specifically addresses adverse reactions that are directly attributable to, or plausibly associated with, hormone therapy based on the available clinical evidence.

### 7.1. Mood

Estradiol plays a key role in brain function through its widespread receptor distribution, influencing memory, neuroprotection, neurogenesis, neurotransmitter regulation, and cerebral metabolism. During menopause, the natural decline in estrogen, alongside shifts in neurosteroids, may disrupt GABAergic balance, contributing to mood instability and emotional dysregulation in perimenopausal women [174,175,176].

However, earlier clinical observations and some studies have also linked hormone replacement therapy to mood fluctuations. Mood fluctuations are especially prevalent in studies on sequential hormone therapy, in which continuous estrogen administration is combined with cyclic progestin. This regimen may elicit adverse mood responses in a subset of postmenopausal women, particularly among those with a prior history of premenstrual symptoms or anxiety-related personality traits [177]. One study found that negative mood symptoms such as irritability, tension, and somatic anxiety during the progestin phase were significantly more common among women with prior premenstrual symptoms and those exhibiting higher baseline scores in anxiety, indirect aggression, and dissatisfaction with life. These findings suggest that individual psychological profiles can influence tolerance to HRT and that the addition of progestin may precipitate mood disturbances in hormonally sensitive women. Careful psychological evaluation and close monitoring are therefore essential when initiating sequential hormonal therapy in patients with known mood vulnerability [177]. Another randomized, double-blind crossover study also demonstrated that higher estradiol exposure during sequential hormone therapy exacerbated progestin-associated adverse effects, including increased psychological symptoms such as irritability, tension, and depressed mood, as well as somatic complaints [178].

In contrast, accumulating recent evidence suggests that when administered appropriately in terms of timing, formulation, and individual patient profile, hormone therapy may in fact exert a stabilizing effect on affective symptoms.

Transdermal estrogen combined with intermittent micronized progesterone was shown to reduce the incidence and severity of depressive symptoms in perimenopausal and early postmenopausal women compared with the placebo. The beneficial mood effects were particularly evident in women in the early menopausal transition and those who had recently experienced stressful life events, highlighting the potential of transdermal HRT as a preventive intervention against mood deterioration during this period [179]. However, the evidence remains controversial, as some studies do not support the same mood-related benefits of transdermal hormone therapy. While other studies have shown that transdermal estrogen combined with progesterone can reduce depressive symptoms in perimenopausal women, one randomized controlled trial (KEEPS-Cog) found different results. In recently postmenopausal women, oral combined hormone therapy (oral conjugated equine estrogens and micronized progesterone) was associated with small to moderate improvements in mood—particularly in depression and anxiety scores—over a 4-year period, whereas transdermal estradiol with the same progestogen showed no significant mood benefit compared to the placebo [180]. These findings suggest that the route of hormone administration may influence psychological outcomes, though the current evidence remains inconclusive regarding the superiority of either approach [180].

Moreover, a randomized placebo-controlled trial found that transdermal estradiol also affected reward-seeking behavior in perimenopausal women [181]. The effect was most evident in those who showed high sensitivity to endogenous estradiol fluctuations and had experienced recent stressful life events. These results suggest that transdermal estrogen may affect not only mood symptoms but also underlying motivational processes, highlighting a potential mechanism by which hormonal changes contribute to altered affective functioning during the menopausal transition. This is particularly relevant as many women in this age group are in the midst of active professional and personal lives, and maintaining motivation and functional capacity is critical for quality of life and productivity [181].

Although both the menopausal transition and hormone replacement therapy, particularly regimens involving certain progestins, have been associated with mood disturbances, current evidence highlights the importance of individualized treatment strategies. Personalized regimens, which take into account a woman’s psychological profile, hormonal sensitivity, and symptom pattern, can help minimize adverse mood effects. Moreover, transdermal estrogen formulations appear to offer a more favorable neuropsychiatric profile compared to oral routes, especially in hormonally sensitive individuals. Tailoring regimens to the needs of each patient may not only reduce the risk of mood swings but also enhance overall quality of life during the menopausal transition.

### 7.2. VTE and Cardiovascular Disease

HRT, as previously stated, has long been associated with an increased risk of thrombotic events, leading to widespread caution in its use. However, this perspective is being increasingly refined as emerging evidence shows that the thrombotic risk is not uniform, but varies significantly depending on several factors, particularly the route of administration and the age at which hormone replacement therapy is initiated.

Standard oral estrogen therapy was associated with increased markers of thrombin generation and reduced levels of natural anticoagulants, indicating an increased risk of venous thromboembolism [182]. Combined oral hormone therapy and a prior history of venous thromboembolism appear to further increase the risk of deep vein thrombosis and pulmonary embolism, as shown in one randomized controlled trial. In this study, women with a documented history of venous thromboembolism who received oral combined hormone replacement therapy (estradiol plus norethisterone acetate) experienced a significantly higher incidence of recurrent thromboembolic events compared to those on the placebo [183]. This finding is supported by the Women’s Health Initiative (WHI) trial, which found a more than twofold increased risk of pulmonary embolism in women receiving combined oral hormone therapy compared to the placebo, with a hazard ratio of 2.13 [184]. This marked elevation in thromboembolic risk contributed to the early termination of the trial due to an unfavorable risk–benefit profile. Collectively, these data underscore the importance of administration route in risk assessment and support the need for caution when prescribing oral combined regimens. These findings support previous observational data and emphasize that oral hormone replacement therapy is associated with a higher risk profile, thus highlighting the need for alternative administration methods [183,184].

Therefore, transdermal formulations were evaluated in search of better risk profiles. Recent studies have demonstrated that transdermal estrogen may carry a much lower thrombotic risk compared to oral formulations, and that initiating HRT closer to the onset of menopause may further reduce cardiovascular and thromboembolic complications.

In a multicenter case–control study of postmenopausal women aged 45 to 70, oral estrogen therapy was associated with a significantly elevated risk of venous thromboembolism (adjusted OR 4.2), while transdermal estrogen use was not linked to an increased venous thromboembolism risk (OR 0.9) [40,41]. These findings further support earlier evidence that the transdermal route is a safer alternative to oral administration in terms of thrombotic complications. Additionally, the study highlighted that not all progestogens carry the same risk: norpregnane derivatives were associated with a substantially increased venous thromboembolism risk, whereas micronized progesterone and pregnane derivatives were not. These distinctions underline the importance of both hormone type and delivery method in minimizing thrombotic risk in hormone therapy [40,41].

The same observations have been made regarding combined hormonal replacement therapy. A large cohort study involving over 950,000 postmenopausal women found no increased risk of venous thromboembolism with current use of transdermal estrogen, either alone or in combination with a progestogen, when compared to non-users. In contrast, oral formulations, both estrogen alone and combined estrogen–progestogen therapy, were associated with a significantly elevated VTE risk, particularly during the first year of use. These findings strongly support the role of the administration route as a major determinant of thrombotic risk in HRT [44].

The data are equally promising for patients with a history of venous thromboembolism. In one long-term follow-up study over 6.5 years involving women with a history of venous thromboembolism, the use of transdermal estrogen hormone therapy was not associated with an increased risk of recurrence, whereas women who used oral estrogen experienced a significantly higher risk, with no subgroups showing elevated recurrence rates with transdermal treatment compared to non-users [185]. Another French multicenter case–control study comparing postmenopausal women with a first episode of idiopathic venous thromboembolism to matched controls found that oral estrogen replacement therapy was associated with a significantly increased risk, with an adjusted odds ratio of 3.5. In contrast, transdermal estrogen therapy showed no significant association with venous thromboembolism, and women using oral estrogen were found to have a fourfold higher risk compared to those using transdermal formulations [43,186].

Moreover, early observational studies suggested that HRT might confer cardiovascular protection and lower mortality rates in postmenopausal women with existing heart disease [187]. However, large randomized clinical trials, including the Women’s Health Initiative and the Heart and Estrogen/Progestin Replacement Studies (HERS I and II), later contradicted these findings, showing that HRT could increase the risk of cardiovascular events [188]. The WHI reported a 30–40% increased risk of stroke in women receiving either combined estrogen–progestin therapy or estrogen alone [189,190]. Despite these concerns, subsequent WHI updates have continued to investigate the long-term cardiovascular effects of both combined conjugated equine estrogens with medroxyprogesterone acetate and conjugated equine estrogens alone [191].

Therefore, this evolving understanding is reshaping clinical recommendations and promoting a more individualized approach to hormone therapy. Although multiple observational and cohort studies suggest a lower thromboembolic and metabolic risk with transdermal formulations, randomized controlled trial data remain limited. Furthermore, residual confounding and population heterogeneity may influence the reported outcomes. Therefore, while current evidence favors transdermal administration in high-risk populations, ongoing debate persists regarding its long-term cardiovascular and thrombotic safety.

### 7.3. Factors for Lack of Efficiency

While the lack of efficacy secondary to various individual factors regarding transdermal hormone replacement therapy has not been widely studied, knowledge on this subject can be drawn from studies on transdermal hormonal therapies for contraceptive purposes. These highlight that efficacy is highly variable based on the degree of obesity, type of hormonal treatment, and study method.

One pooled analysis of three clinical trials showed that the contraceptive patch (Ortho Evra/Evra) is a highly effective birth control method with good cycle control across diverse groups of women. The rate of unintended pregnancies was low and bleeding patterns improved over time. For women under 90 kg, contraceptive efficacy remained consistently high regardless of weight. However, there may be a reduced level of effectiveness in women weighing 90 kg or more. Overall, the patch demonstrated reliable contraceptive efficacy and bleeding control comparable to traditional combined oral contraceptives [192].

Another study showed similar results and aimed to compare the safety and tolerability of a new low-dose contraceptive patch containing levonorgestrel and ethinyl estradiol with two types of combination oral contraceptives in women aged 17 to 40. Conducted across two Phase 3 clinical trials, the research included a diverse population, with around 30% of participants classified as obese. Adverse events such as nasopharyngitis, nausea, headache, and skin reactions were reported at similar rates across all treatment groups. Importantly, the incidence of adverse events did not differ significantly between obese and non-obese women. Serious adverse events were rare in all groups. Overall, the contraceptive patch was well-tolerated and showed a safety profile comparable to that of traditional oral contraceptives, regardless of body weight.

Therefore, further research is needed on the tolerability and efficacy of hormone replacement therapy in individuals with obesity. However, existing evidence from similar transdermal hormonal patches used for contraception shows promising results, with comparable outcomes across all weight groups [109].

## 8. Future Perspectives and Long-Term Safety Considerations

Future perspectives in menopausal hormone therapy are evolving, focusing on optimizing safety and efficacy. Current research underscores the importance of the “timing hypothesis”, which suggests that initiating therapy near the onset of menopause, particularly before the age of 60, may confer cardiovascular benefits without increasing associated risks. This approach aims to enhance the protective effects of estrogen on cardiovascular health while mitigating potential adverse outcomes [193].

Additionally, ongoing studies are examining the impact of different formulations and delivery methods on cardiovascular risk profiles. For instance, transdermal estradiol has been associated with a more favorable cardiovascular risk profile compared to oral conjugated equine estrogens, possibly due to differences in hepatic metabolism and coagulation factor modulation. These findings are guiding the development of personalized regimens that consider individual risk factors and preferences [194].

On the other hand, future directions increasingly emphasize the role of hormonal therapy in maintaining skin integrity and mitigating age-related dermal changes. Estrogen deficiency during menopause contributes to decreased collagen content, reduced skin elasticity, impaired hydration, and slower wound healing, which collectively accelerate cutaneous aging. Emerging research suggests that tailored regimens, particularly using transdermal estradiol, can improve dermal thickness, elasticity, and hydration, while potentially reducing wrinkle formation. Future investigations are likely to focus on optimizing dosage, timing, and formulation for individualized cutaneous benefits, assessing long-term safety, and exploring synergistic effects with topical cosmeceuticals. Additionally, studies are needed to understand the interplay between menopausal hormone therapy and skin cancer risk, given estrogen’s influence on melanocytes and keratinocytes, aiming to develop evidence-based protocols for postmenopausal women seeking dermatologic as well as systemic benefits [195,196].

Current clinical guidelines recommend prescribing menopausal hormone therapy at the lowest effective dose for the shortest duration necessary to achieve symptom control, with regular reassessment of individual benefit–risk profiles [30,31]. Evidence regarding prolonged use beyond 5–10 years is limited and heterogeneous. Observational and randomized studies, including long-term follow-up from the Women’s Health Initiative, suggest that extended therapy may be associated with increased risks of breast cancer, venous thromboembolism, and stroke in certain populations [42,161,184,190,191]. Therefore, long-term use of MHT should be individualized, periodically reviewed, and based on shared decision-making between clinician and patient.

In summary, future advancements in menopausal hormone therapy aim to refine treatment strategies by considering the timing of initiation, formulation types, and delivery methods to optimize health outcomes in postmenopausal women.

## 9. Methodological Limitations of Available Evidence

The interpretation of current evidence on transdermal menopausal hormone therapy is limited by substantial heterogeneity among published studies. Variability in study design, population characteristics, hormone formulations, dosing regimens, outcome measures, and duration of follow-up complicates direct comparisons and meta-analytic synthesis.

Additionally, many studies are observational in nature or include relatively short follow-up periods, restricting the assessment of long-term safety and efficacy. These methodological limitations should be considered when interpreting the reported benefits and risks.

## 10. Conclusions

Menopausal hormone therapy remains central in the management of symptoms and the preservation of women’s long-term health. Over recent years, the development of alternative delivery routes has redefined the therapeutic landscape, with transdermal formulations gaining particular relevance due to their favorable pharmacological and safety profiles.

This review synthesizes and clarifies existing evidence on the clinical role of transdermal hormone therapy, highlighting its advantages compared with conventional oral administration. Current data consistently demonstrate that transdermal delivery maintains stable systemic estradiol concentrations while reducing thromboembolic and metabolic risks associated with hepatic first-pass metabolism. The analysis further underscores the versatility of transdermal systems, including patches, gels, emulsions, and sprays, along with their suitability for individualized therapy. The addition of progestogens, whether in fixed combinations or via intrauterine levonorgestrel systems, provides reliable endometrial protection in women with an intact uterus. Moreover, the combined use of 17β-estradiol and norethisterone acetate has been associated with beneficial modulation of cardiovascular risk parameters, while restoration of near-physiological hormone levels may contribute to improved dermal hydration, collagen content, and elasticity.

Despite these strengths, this review also identifies persistent knowledge gaps. Evidence remains inconsistent regarding the pharmacokinetics of progesterone, the relationship between serum and tissue hormone levels and the cognitive and dermatologic outcomes (including skin cancer development) of long-term use. Furthermore, standardized criteria for dosing, formulation selection, and outcome assessment are still lacking across studies.

In conclusion, transdermal hormone therapy emerges as an effective, safe, and physiologically coherent approach to menopausal care, with clear benefits over oral formulations. Continued high-quality research is essential to refine dosing strategies and consolidate its role within a personalized and evidence-based framework for managing postmenopausal health.

## Figures and Tables

**Table 1:** Comparative pharmacological characteristics of bioidentical and synthetic hormones used in menopausal hormone therapy.

Feature	Bioidentical Hormones	Synthetic Hormones
Molecular structure	Identical to endogenous human hormones	Structurally modified molecules
Receptor affinity	Mimic physiological receptor binding	May exhibit variable receptor interactions
Metabolism	Follows physiological metabolic pathways	Greater variability due to structural modifications
Hepatic metabolism	Less pronounced when non-oral routes are used	More influenced by hepatic first-pass metabolism
Pharmacokinetics	More closely resemble endogenous hormone profiles	May differ from physiological hormone dynamics
Clinical use considerations	Often used in individualized treatment approaches	Widely used with established clinical data

**Table 2:** Key differences between oral and transdermal HRT. In the table, ↑ indicates an increase and ↓ indicates a decrease.

Feature	Oral HRT	Transdermal HRT	Supporting Evidence	Reference
First-pass metabolism	Present	Absent	Hepatic metabolism influences coagulation factors and lipid profile	Castelo-Branco et al., 2014 [108], Vaisar et al., 2021 [113]
Serum hormone stability	Moderate	High	Transdermal delivery provides more stable serum hormone levels	Vaisar et al., 2021 [113]
Thromboembolic risk	Higher	Lower	Oral route associated with increased thromboembolic risk due to hepatic effects	Castelo-Branco et al., 2014 [108]
Lipid profile effects	Variable (↑ TG, HDL changes)	Neutral/favorable (↓ LDL, stable TG)	Transdermal estrogen reduces LDL without increasing triglycerides	Vaisar et al., 2021 [113]
Dose flexibility	Limited	High	Transdermal formulations (patch, gel, spray) allow flexible dosing	Kovács et al., 2016 [111]
Suitability for high-risk patients	Limited	Preferred	Transdermal route preferred in patients with metabolic or cardiovascular risk factors	Castelo-Branco et al., 2014 [108], Vaisar et al., 2021 [113]
Gastrointestinal effects	Possible	Rare	Oral administration associated with GI exposure and hepatic metabolism	Castelo-Branco et al., 2014 [108]

**Table 3:** Drug interactions of HRT and current evidence level.

Drug Class/Clinical Aspect	Mechanism of Interaction	Route Affected	Clinical Consequence	Reference
Thyroid hormone replacement therapy	Oral estrogens increase thyroxine-binding globulin (TBG), reducing free thyroid hormone levels	Oral	May require adjustment of thyroid hormone dosage	Mazer et al., 2004 [172]
Cytochrome P450-metabolized drugs (e.g., carbamazepine, rifampin)	Hepatic enzyme modulation affecting drug metabolism	Oral	Potential alteration of drug efficacy	Valdes et al., 2025 [173]
Coagulation-related medications	Estrogens influence coagulation parameters	Oral > Transdermal	Possible interaction affecting bleeding/thrombotic risk	Fasero et al., 2023 [171]
Multiple pharmacological groups (23 classes identified)	Variable interaction potential depending on drug class and formulation	Oral and Transdermal	Requires individualized evaluation; vaginal forms show minimal interaction risk	Valdes et al., 2025 [173]

## Data Availability

No new data were created or analyzed in this study.

## Abbreviations

The following abbreviations are used in this manuscript:

PCOS	Polycystic Ovary Syndrome
FSH	Follicle Stimulating Hormone
LH	Luteinizing Hormone
VTE	Venous Thromboembolism
EE	Ethinyl Estradiol
E2	17β-Estradiol
E2V	Estradiol Valerate
E4	Estetrol
NLCs	Nanostructured Lipid Carriers
SLNs	Solid Lipid Nanoparticles
SHBG	Sex Hormone-Binding Globulin
DHT	Dihydrotestosterone
HSDD	Hypoactive Sexual Desire Disorder
LNG-IUS	Levonorgestrel-Releasing Intrauterine Device
IUD	Intrauterine Contraceptive Device
HRT	Hormone Replacement Therapy
MENQOL	Menopause-Specific Quality of Life
MHT	Menopausal Hormone Therapy
AD	Alzheimer’s Disease
WHISCA	WHI Study of Cognitive Aging
ERs	Estrogen Receptors
WHI	Women’s Health Initiative
